# Protein-Bound Uremic Toxins Lowering Effect of Sevelamer in Pre-Dialysis Chronic Kidney Disease Patients with Hyperphosphatemia: A Randomized Controlled Trial

**DOI:** 10.3390/toxins13100688

**Published:** 2021-09-27

**Authors:** Kullaya Takkavatakarn, Pongpratch Puapatanakul, Jeerath Phannajit, Warumphon Sukkumme, Pajaree Chariyavilaskul, Patita Sitticharoenchai, Asada Leelahavanichkul, Pisut Katavetin, Kearkiat Praditpornsilpa, Somchai Eiam-Ong, Paweena Susantitaphong

**Affiliations:** 1 Division of Nephrology, Department of Medicine, Faculty of Medicine, King Chulalongkorn Memorial Hospital, Chulalongkorn University, Bangkok 10330, Thailand; kullaya.t@chula.ac.th (K.T.); pongpratch.p@chula.ac.th (P.P.); jeerath.p@chula.ac.th (J.P.); asada.l@chula.ac.th (A.L.); pisut.k@chula.ac.th (P.K.); kearkiat.p@chula.ac.th (K.P.); somchai.e@chula.ac.th (S.E.-O.); 2 Clinical Pharmacokinetics and Pharmacogenomics Research Unit, Department of Pharmacology, Faculty of Medicine, Chulalongkorn University, Bangkok 10330, Thailand; varum_sk@hotmail.com (W.S.); pajaree.l@chula.ac.th (P.C.); 3 Department of Pharmacology, Faculty of Medicine, Chulalongkorn University, Bangkok 10330, Thailand; 4 Division of Cardiology, Department of Medicine, Faculty of Medicine, King Chulalongkorn Memorial Hospital, Chulalongkorn University, Bangkok 10330, Thailand; patita.s@chula.ac.th; 5 Department of Microbiology, King Chulalongkorn Memorial Hospital, Thai Red Cross Society, Bangkok 10330, Thailand; 6 Translational Research in Inflammation and Immunology Research Unit (TRIRU), Department of Microbiology, Chulalongkorn University, Bangkok 10330, Thailand; 7 Research Unit for Metabolic Bone Disease in CKD Patients, Faculty of Medicine, Chulalongkorn University, Bangkok 10330, Thailand

**Keywords:** p-cresyl sulfate, indoxyl sulfate, protein-bound uremic toxins, sevelamer, chronic kidney disease

## Abstract

P-cresyl sulfate and indoxyl sulfate are strongly associated with cardiovascular events and all-cause mortality in chronic kidney disease (CKD). This randomized controlled trial was conducted to compare the effects between sevelamer and calcium carbonate on protein-bound uremic toxins in pre-dialysis CKD patients with hyperphosphatemia. Forty pre-dialysis CKD patients with persistent hyperphosphatemia were randomly assigned to receive either 2400 mg of sevelamer daily or 1500 mg of calcium carbonate daily for 24 weeks. A significant decrease of total serum p-cresyl sulfate was observed in sevelamer therapy compared to calcium carbonate therapy (mean difference between two groups −5.61 mg/L; 95% CI −11.01 to −0.27 mg/L; *p* = 0.04). There was no significant difference in serum indoxyl sulfate levels (*p* = 0.36). Sevelamer had effects in terms of lowering fibroblast growth factor 23 (*p* = 0.01) and low-density lipoprotein cholesterol levels (*p* = 0.04). Sevelamer showed benefits in terms of retarding CKD progression. Changes in vascular stiffness were not found in this study.

## 1. Introduction

Chronic kidney disease (CKD) is one of the most important global health problems, and it significantly increases cardiovascular risk and mortality [1]. Several risk factors for CKD have been proposed, including traditional risk factors, such as diabetes, hypertension, and dyslipidemia, and uremia-related risk factors including anemia, mineral and bone disorders, inflammation, oxidative stress, and uremic toxins [2].

As renal function declines, phosphate excretion is increased by reducing the tubular reabsorption of the filtered phosphate in the remaining nephrons under the influence of fibroblast growth factor 23 (FGF23) and the parathyroid hormone (PTH) to maintain normal serum phosphate levels. In stage 4–5 CKD, adaptation is no longer adequate, and hyperphosphatemia develops despite high FGF23 levels [3]. The very high levels of FGF23 permit anomalous FGF receptor activation independent of Klotho, resulting in unique FGF23-stimulated pathologies. Several experimental and epidemiological studies in CKD have illustrated correlations between high FGF23 levels and various deleterious effects, including left ventricular hypertrophy [4], vascular calcification [5], impaired immune system, anemia, and decreased bone mineralization [6].

P-cresyl sulfate and indoxyl sulfate are the two most studied protein-bound uremic toxins [7]. Both substances originate from the amino acid metabolism of colonic bacteria. Chronically heightened levels of p-cresyl sulfate and indoxyl sulfate are associated with multiple adverse outcomes, including the enhanced progression of renal failure, increased cardiovascular disorders and mortality, and elevated all-cause mortality in both pre-dialysis and dialysis CKD [8]. Because of their binding affinity to albumin, protein-bound uremic toxins cannot be effectively removed by conventional hemodialysis or peritoneal dialysis. Certain non-dialysis strategies such as a protein-restricted diet and manipulation of the gut microbiota have been conducted to lower serum p-cresyl sulfate and indoxyl sulfate levels [9]. However, effective therapeutic reduction strategies for these toxins are still limited.

Sevelamer, an anion exchange resin, is a widely used non-calcium-based phosphate binder that can bind other anionic substances. In addition to the phosphate-binding effect, sevelamer seems to exert pleiotropic effects, including lipid profile and inflammatory marker improvements. A previous in vitro study showed that sevelamer could directly sequester p-cresol, a precursor of p-cresyl sulfate [10]. A small cohort study revealed a significant reduction of serum p-cresyl sulfate in hemodialysis patients [11]. There have been two previous RCTs that investigated the effect of sevelamer on serum p-cresyl sulfate reduction in pre-dialysis CKD patients with normophosphatemia, and these RCTs reported conflicting results [12,13]. With regard to serum indoxyl sulfate reduction, an in vitro study demonstrated that sevelamer hydrochloride binds indole by 10–15% [10]. However, in a previous observational study in five hemodialysis patients, sevelamer did not reduce serum indoxyl sulfate levels after 12 weeks [11]. In addition, a recent post hoc study illustrated that serum indoxyl sulfate was not altered in pre-dialysis CKD patients receiving sevelamer for 12 weeks [13].

This is the first randomized controlled trial (RCT) to examine the serum p-cresyl sulfate and indoxyl sulfate lowering effects of sevelamer compared to calcium-based phosphate binders in pre-dialysis CKD patients with hyperphosphatemia.

## 2. Results

### 2.1. Patients Characteristics

**Figure 1:**
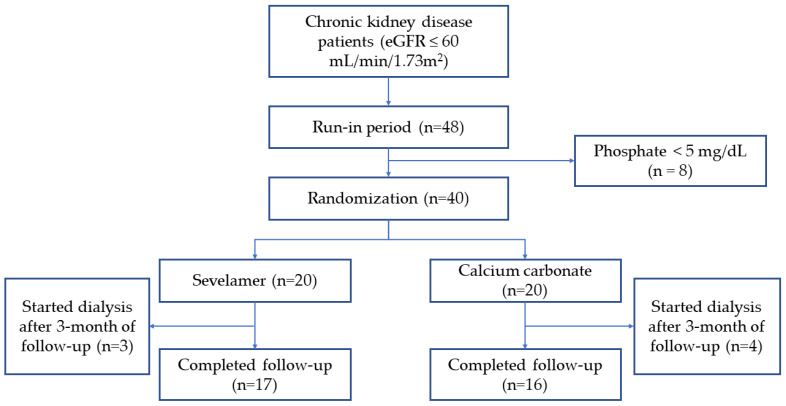
Study flow chart.

Among 250 pre-dialysis CKD patients in the outpatient department, 48 patients were eligible for inclusion in this trial. Forty patients with persistent hyperphosphatemia after the run-in period were randomized to receive sevelamer (n = 20) or calcium carbonate (n = 20). After a 12-week follow-up visit, seven patients started renal replacement therapy. Thirty-three patients completed the study (Figure 1). At randomization, the mean serum phosphate levels were 5.50 ± 0.17 and 5.39 ± 0.10 mg/dL in the sevelamer and calcium carbonate groups, respectively. The serum p-cresyl sulfate levels were 10.75 ± 2.20 and 8.75 ± 1.26 mg/L while the serum indoxyl sulfate levels were 17.97 ± 2.55 mg/L and 16.16 ± 2.49 mg/L in the sevelamer and calcium carbonate groups, respectively (Table 1).

Most of the patients were CKD stage 5 (90%). Diabetic nephropathy was the most common cause of CKD in both groups. There were no differences between the two groups at baseline in terms of demographic data, renal function, laboratory parameters, CAVI, left ventricular mass index (LVMI), and left ventricular ejection fraction (LVEF). Fifty-five percent of the patients had CAVI > 8. More than 80 percent of the patients in both groups were diagnosed with left ventricular hypertrophy (Table 1).

### 2.2. Changes in Serum P-cresyl Sulfate Concentration

**Figure 2:**
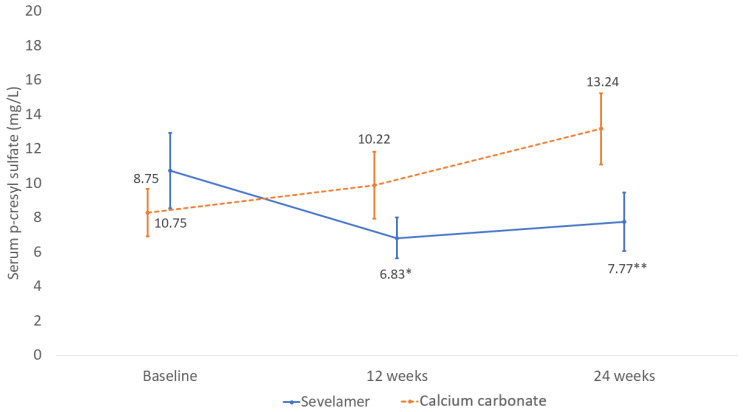
Mean change serum p-cresyl sulfate levels in the sevelamer and calcium carbonate groups during the 24-week follow-up period. Data shown as mean ± SE (* significant difference within a group with respect to baseline by using linear mixed model; ** significant difference between groups).

When the serum p-cresyl sulfate reductions were compared between the sevelamer and calcium carbonate groups at the 24-week follow-up, there was a significant reduction in the sevelamer group (mean difference between the two groups −5.61 mg/L; 95% CI −11.01 to −0.27 mg/L; *p* = 0.04) (Figure 2).

In addition, the serum p-cresyl sulfate levels were significantly decreased from those at baseline in the sevelamer group (*p* = 0.01) but were unaltered in the calcium carbonate group (*p* = 0.08).

### 2.3. Changes in Serum Indoxyl Sulfate Concentration

**Figure 3:**
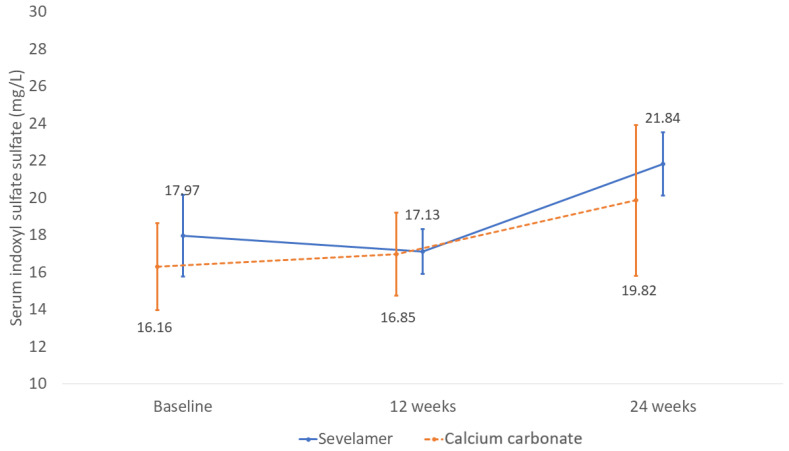
Mean change of serum indoxyl sulfate levels in the sevelamer and calcium carbonate groups during the 24-week follow-up period. Data shown as mean ± SE.

The changes of the serum indoxyl sulfate levels between sevelamer and calcium groups during follow-up of were not statistically different (mean difference between the two groups 2.31 mg/L; 95% CI −10.25 to 14.88 mg/L; *p* = 0.36) (Figure 3).

At the 24-week follow-up, there were no significant differences in the serum indoxyl sulfate levels from those at baseline in both the sevelamer (*p* = 0.40) and calcium carbonate groups (*p* = 0.49).

### 2.4. Changes in Serum Calcium Phosphate, PTH, and FGF 23

There were no significant differences in the serum calcium, phosphate, and PTH levels during the study period between the sevelamer and calcium carbonate treatment groups (Table 2). Interestingly, the sevelamer group had a significant change in their FGF23 levels from baseline when compared to the calcium carbonate group at 24 weeks (*p* = 0.01) (Table 2).

Using the Wilcoxon signed-rank test, there was no significant change in the FGF23 levels from baseline to the 24-week follow-up in the sevelamer group. The median (IQR) values were 47.19 (91.08) and 57.20 (122.27) pg/mL, respectively (*p* = 0.58). However, the FGF23 levels increased significantly from baseline to the 24-week follow-up in the calcium carbonate group. The median (IQR) levels were 61.50 (83.73) and 106.07 (208.40) pg/mL, respectively, *p* = 0.04).

### 2.5. Effects on Renal Function and Proteinuria

There were no significant differences in the renal function changes (mean difference between group −0.02; 95% CI −5.17 to 5.13 mL/min/1.73m^2^; *p* = 0.99) and proteinuria (mean difference between group −0.17; 95% CI −1.37 to 1.02 g/day; *p* = 0.78) between the patients treated with sevelamer and calcium carbonate (Table 2).

During the 24-week follow-up, the patients in both groups had significant renal function reductions (*p* = 0.04 in the sevelamer group and *p* = 0.001 in the calcium carbonate group). In terms of dialysis initiation, three patients in the sevelamer group and four patients in the calcium carbonate group initiated dialysis during the study. There was no significant difference in terms of the cumulative incidence of dialysis initiation between the sevelamer and calcium carbonate groups (hazard ratio 0.64 (95% CI 0.14 to 2.87; *p* = 0.56).

### 2.6. Effects on Low-Density Lipoprotein (LDL) Cholesterol

There was a significant difference in the LDL-cholesterol changes from baseline between the patients treated with sevelamer and calcium carbonate (mean difference between group −26.2 mg/dL; 95% CI −40.5 to −11.89 mg/dL; *p* = 0.04).

At the 24-week follow-up, the sevelamer group had a significant reduction of their LDL-cholesterol levels from baseline (*p* < 0.001), whereas there were no significant changes in the serum LDL-cholesterol levels after receiving calcium carbonate (*p* = 0.40) (Table 2).

### 2.7. Effects on Inflammatory Markers (hs-CRP Levels)

At the end of treatment, no significant changes in the hs-CRP levels between the sevelamer and calcium carbonate groups were demonstrated (*p* = 0.64).

### 2.8. Effects on Vascular Stiffness and Peripheral Arterial Disease

After the 24-week follow-up, there were no significant changes in the CAVI and ABI between the sevelamer and calcium carbonate groups (mean difference −0.1, 95%CI −0.35 to 0.15; *p* = 0.42 and −0.014; 95% CI −0.06 to 0.04; *p* = 0.57, respectively).

### 2.9. Follow-Up Data

All of the patients had good adherence to the provided dietary advice. The mean DPI ranged from 0.68 to 0.72 g/kg/day during the study period. More than 90 percent of the patients had excellent medication adherence according to pill count. 

No patient dropped out after randomization. Seven patients (17.5%), three of whom were initially assigned to the sevelamer group and four of whom were in the calcium carbonate group, indicated renal replacement therapy during the study. All of the patients started dialysis after a 12-week follow-up visit where they deisplayed certain indications (five patients developed poor appetite and nausea, and two patients had clinical volume overload). There was no emergency dialysis initiation. No patients had a cardiovascular event or died during the follow-up period.

### 2.10. Adverse Events

One modest gastrointestinal side effect was reported in a patient receiving sevelamer. None of the patients had hypophosphatemia or hypercalcemia in either group.

## 3. Discussion

In this study, we explored the lowering effects of sevelamer, an anion exchange resin that is a widely used non-calcium-based phosphate binder, on the levels of p-cresyl sulfate, indoxyl sulfate, and various uremic toxins. The study was performed in pre-dialysis CKD patients with hyperphosphatemia and was compared to calcium carbonate, another phosphate-binding agent. This RCT is the first study to demonstrate the lowering effects of sevelamer on the serum p-cresyl sulfate level compared to calcium carbonate therapy. Moreover, sevelamer was shown to be able to lower LDL-cholesterol and FGF23 levels compared to calcium carbonate. However, there were no significant differences in the serum indoxyl sulfate and hs-CRP levels after treatment.

The human intestine is known as the habitat of more than 100 trillion micro-organisms that provide several metabolic products and allocate the human immune system. There are both qualitative and quantitative alterations in the composition of the gut microbiota in CKD patients due to the decreased consumption of dietary fibers, the use of multiple drugs, metabolic acidosis, intestinal wall edema, and the accumulation of uremic toxins [14]. The higher number of pathogenic microbes and the increased intestinal permeability of CKD patients contribute to the elevation of gut-derived uremic toxins such as p-cresyl sulfate and indoxyl sulfate [15]. Therefore, the increased serum p-cresyl sulfate levels in CKD patients result from decreased renal excretion and increased production due to the changes in the intestinal microbiome, which promote the production of these compounds.

Although a previous in vitro study showed the binding ability of sevelamer to the precursor of p-cresyl sulfate, the lowering effect was not observed in the CKD mouse model. Only a few clinical studies have evaluated the impact of sevelamer on uremic toxins. Previous RCTs investigating the effect of sevelamer regarding this issue yielded conflicting outcomes. Riccio et al. showed that 1600 mg of sevelamer carbonate effectively reduced serum p-cresyl sulfate in pre-dialysis CKD stage 3–5 patients compared to a placebo after 12 weeks of treatment [12]. In comparison, Bennis et al. illustrated an non-significant association between a 12-week treatment with 4800 mg of sevelamer carbonate per day and serum p-cresyl sulfate and indoxyl sulfate changes in CKD stage 3b and 4 patients compared to a placebo [13]. These different results might be able to be explained by the disparities in the study populations, baseline renal function, and serum p-cresyl sulfate level, and follow-up time.

Our study, which was the first to compare sevelamer with a calcium-based phosphate binder, was performed in very advanced pre-dialysis CKD patients. Ninety percent of our patients were CKD stage 5, and the mean eGFR was 10.4 mL/min/1.73 m^2^, which was obviously lower than previous studies (mean eGFR was 38.7 and 27 mL/min/1.73 m^2^) [12,13]. The different baseline renal function resulted from the inclusion criteria of hyperphosphatemia in our study. Early stage CKD patients could control their serum phosphate levels to within the normal range by following a low-protein and -phosphate diet. Furthermore, despite the declined renal function, patients with early stage CKD were able to enhance their renal phosphate excretion by means of increased FGF23 production. Therefore, pre-dialysis CKD patients usually only develop hyperphosphatemia when they have late stage 4 or stage 5 CKD [16]. As our patients were advanced CKD patients, the baseline serum p-cresyl sulfate was higher than it was in the previous study [12]. Moreover, in this study, the patients received intervention and were followed up with for 24 weeks, which was longer than the follow-up period in earlier reports. According to our results, the reduction of serum p-cresyl sulfate in the sevelamer group was initially observed at 12 weeks; however, a significant difference between the two groups was revealed at 24 weeks (Figure 1). More impaired renal function with a longer follow-up period might explain the positive effects of sevelamer that were observed in our study.

We also explored the lowering effects of sevelamer on other uremic toxins and substances, including indoxyl sulfate, FGF23, and hs-CRP. We found no significant change in the serum indoxyl sulfate levels after receiving sevelamer or calcium carbonate treatment. These results were supported by previous studies. In an early in vitro study, Bennis et al. revealed that sevelamer did not have the ability to chelate indole, the precursor of indoxyl sulfate, regardless of the pH value [13]. A cross-over interventional study by Brandenburg et al. investigated the effect of sevelamer on the serum indoxyl sulfate level in 41 hemodialysis patients. After 8 weeks of sevelamer hydrochloride and calcium acetate treatment, there was no significant changes in serum indoxyl sulfate concentration [17]. Very recently, a multicenter, double-blind, placebo-controlled, RCT reported an non-significant indoxyl sulfate reduction after sevelamer treatment in 78 pre-dialysis CKD patients [13].

FGF23 was another uremic toxin that could be effectively reduced by sevelamer in this study. In addition, this effect was independently associated with phosphate levels. The primary function of FGF23 in CKD patients is to enhance renal phosphate excretion. The FGF23 level continuously rises during the progression of CKD in order to maintain a normal phosphate level. Several studies have illustrated correlations between high FGF23 levels and numerous deleterious effects, including left ventricular hypertrophy, vascular calcification, and mortality [18,19]. As phosphate is an important stimulating factor for FGF23 production, phosphate binders are expected to be an efficacious FGF23 lowering modality. However, previous data have shown that not all phosphate binders can reduce FGF23 levels [20]. A recent systematic review and meta-analysis supported the positive effect of sevelamer on FGF23 in our study. Takkavatakarn et al. reported a significant decrease of FGF23 in CKD patients receiving sevelamer compared to either calcium carbonate or a placebo [20]. In contrast, there was no significant reduction in the FGF23 levels of patients treated with lanthanum, another non-calcium-based phosphate binder when compared to the placebo group. These findings demonstrate that the effect of sevelamer on FGF23 reduction could not be explained by the phosphate lowering ability of phosphate binders but might result from the pleiotropic effects of sevelamer. In addition, the calcium supplementation from the calcium-based phosphate binder might stimulate FGF23 production.

Previous data showed the potency of sevelamer in diminishing endotoxin, a glycolipid component of the Gram-negative cell wall that is a potent stimulus for proinflammatory cytokine production [21]. In advanced CKD, bacterial translocation from the gastrointestinal tract is a significant source of endotoxins [22]. According to its negative charge properties, sevelamer could effectively sequester endotoxins in both in vitro and in vivo studies [23]. A previous observational study showed that sevelamer could lessen endotoxin levels and consequently decrease proinflammatory cytokines and the C-reactive protein in hemodialysis patients [24]. However, the reduction of hs-CRP was not observed in our study. A possible explanation for the discrepancy among the studies might be the differences in the study population and the baseline hs-CRP levels of the patients. Indeed, systemic inflammation increases as renal function progressively declines. The pathophysiology involved in intensifying chronic inflammation has been described as multifactorial factors, including alteration of the gut microbiota, intravenous iron supplements, and the retention of several uremic toxins [25]. Previous studies that observed the reduction of hs-CRP after sevelamer treatment only performed in maintenance hemodialysis patients with high hs-CRP levels. In contrast, a recent RCT that explored this outcome in pre-dialysis CKD patients did not demonstrate any significant differences between the sevelamer and control groups [12]. In our study, the patients in the sevelamer and calcium carbonate groups had worsening renal function and increasing hs-CRP levels compared to baseline. Therefore, the effects of sevelamer on the changes in the hs-CRP levels may not be demonstrated. However, it should be noted that our sample size may be too small to detect any differences in terms of secondary outcomes. 

We also evaluated renal outcomes, including renal progression and incidence of dialysis initiation. There were no significant differences in the renal outcomes between the sevelamer and calcium carbonate groups. Since ninety percent of the participants in this study had stage 5 CKD with the mean eGFR of about 10 mL/min/1.73m^2^, it could have been too late to manipulate renal progression. 

P-cresyl sulfate is known as a vascular toxin since it induces inflammation and oxidative stress via leukocyte activation, causing vascular endothelial injury and the reduction of nitric oxide production [26,27,28]. A previous systematic review of 27 studies both in vitro and in vivo reported the positive correlations between serum p-cresyl sulfate levels and vascular dysfunction together with aortic calcification [7]. The p-cresyl sulfate level was also significantly associated with cardiovascular events and all-cause mortality in CKD patients [8]. The reduction of serum p-cresyl sulfate is expected to be a promising strategy to improve cardiovascular outcomes, which are responsible for being a major cause of death of CKD patients. The clinical trial focusing on long-term clinical outcomes in pre-dialysis CKD was lacking. Previous prospective studies in hemodialysis patients reported that sevelamer could suppress vascular and valvular calcification progression compared to calcium-based phosphate-binders after 12 months of follow-up [29,30]. This study measured vascular stiffness caused by CAVI and ABI at baseline and at the end of the study; however, no significant vascular stiffness changes were observed in any of the patients. These findings might be limited by study duration. A longer-term follow-up study targeting vascular outcomes is still warranted.

This is the first RCT focusing on the effect of sevelamer on serum p-cresyl sulfate reduction in very advanced-stage pre-dialysis CKD patients with hyperphosphatemia who had been recommended phosphate binders according to current guidelines. We provided novel advantages of sevelamer over calcium-based phosphate binders, including lower serum p-cresyl sulfate and FGF23 levels. Participants demonstrated good compliance with dietary counseling as well as well-controlled protein and phosphate intake throughout the study period, which are important factors influencing gut-derived uremic toxin production. In addition, intervention adherence was excellent, as determined by pill counts.

There are some limitations in this study. First, the number of participants is relatively small. Although we performed appropriate statistical calculations and the primary outcome could achieve statistical significance, the power of analysis of the secondary outcomes was limited. The potency of sevelamer on lowering other substances such as indoxyl sulfate requires further study. Second, our study was conducted in advanced stage CKD patients and did not represent a long-term follow-up period. Thus, we may not have detected the effect of sevelamer on clinical outcomes, including vascular stiffness and cardiovascular events. Further studies on the effect of sevelamer on cardiovascular changes in earlier-stage CKD with a longer follow-up period are still required.

## 4. Conclusions

In conclusion, sevelamer could effectively reduce serum p-cresyl sulfate levels and lipid profiles in pre-dialysis CKD patients with hyperphosphatemia. The differences in the serum indoxyl sulfate level, renal progression, and dialysis initiation were not observed between the sevelamer and calcium carbonate treatment groups. Our data suggest additional benefits of sevelamer over calcium-based phosphate binders in reducing cardiovascular toxic substances in CKD patients.

## 5. Methods

### 5.1. Study Population

This single-center RCT was conducted on CKD patients from July 2018 to December 2020. The inclusion criteria were patients who were older than 18-years-old and who had pre-dialysis CKD with an estimated glomerular filtration rate (eGFR) < 60 mL/min/1.73 m^2^ as defined by the Chronic Kidney Disease Epidemiology Collaboration (CKD-EPI) equation [31] for at least 12 weeks with hyperphosphatemia (>5.0 mg/dL). Exclusion criteria were existing or previous treatment with a phosphate binder within the last month, recently adjusted doses of vitamin D analogue within the last 3 months, patients with a serum calcium level that was more than 10.2 mg/dL, and pregnancy.

This study was approved by The Research Ethics Review Committee for Research Involving Human Research Participants, Health Sciences Group, Chulalongkorn University. The study was registered with the Thai Clinical Trials Registry (TCTR20181018003).

### 5.2. Study Design and Procedures

We conducted a prospective, open-label, RCT. During the 2 weeks of the run-in period, all of the patients were given dietary advice for CKD, including a low-protein (0.6–0.8 g/kg/day), low-salt (sodium < 2 g per day), and low-phosphate diet (<800 mg per day) from dietitians. Patients who had persistent hyperphosphatemia at the end of the run-in period were randomly assigned to receive either daily sevelamer or calcium carbonate. 

This study used sevelamer carbonate (Renvela^®^) 800 mg per tablet or calcium carbonate 1000 mg per tablet (containing calcium element for 400 mg per tablet). All patients received 2400 mg of sevelamer (one tablet three times daily with meals) or 1500 mg of calcium carbonate (half a tablet three times daily with meals) for 24 weeks. Drug dosage was adjusted according to serum phosphate level after 6 weeks of treatment. Dosage was reduced by 50% in patients who had hypophosphatemia (<2.5 mg/dL), and serum phosphate was measured in the 4 weeks after dose adjustment. Medication compliance was assessed using pill counts.

Concomitant pharmacological and non-pharmacological therapies such as renin-angiotensin-aldosterone blockage, anemia management, and blood pressure control were prescribed to all patients according to the therapeutic target of standard guidelines [32]. Dietary advice and a dietary diary were organized by the dietitians throughout the study period.

Withdrawal from the study was considered in cases of drug intolerance, persistent hypophosphatemia (<2.5 mg/dL) or hypercalcemia (>10.2 mg/dL) on two sequential blood tests, and the presentation of an indication to initiate renal replacement therapy. Data before patient withdrawal were included in the analyses.

### 5.3. Data Collection

Demographic data, including age, sex, causes of CKD, and medical history were obtained. Echocardiography at baseline was evaluated by a cardiologist in a blinded performance. Ankle–brachial index (ABI) and cardio–ankle vascular index (CAVI) were used to determine vascular complications at baseline and 24 weeks.

At each time period of the study (baseline, 6, 12, and 24 weeks), a complete clinical evaluation, including body weight and blood pressure measurement, was performed. The biochemical parameters were obtained. Urinary urea nitrogen, creatinine, sodium, phosphate, and protein excretion were measured in 24-hour urine. Dietary protein intake (DPI) was estimated by daily urinary excretion of urea nitrogen. Renal function was expressed as eGFR, which was calculated by the CKD-EPI equation. All tests were determined at the central laboratory using standardized procedures.

Serum total p-cresyl sulfate, total indoxyl sulfate, FGF23, PTH, lipid profile, and hs-CRP were measured at baseline, 12, and 24 weeks. The total p-cresyl sulfate and indoxyl sulfate serum levels were determined by high-performance liquid chromatography (HPLC) at the Pharmacology Department, Faculty of Medicine, Chulalongkorn University. The average intra-assay coefficient of variation (CV) of HPLC was 1.65%. The laboratory technician was blinded to the treatment assignment. The PTH levels were measured using a chemiluminescence immunoassay on a Roche Elecsys 2010 Analyzer. This assay detected both intact PTH and a fragment containing amino acids 7 to 84. The FGF23 levels were assessed using a human intact FGF23 ELISA kit (Millipore Corporation, Billerica, MA, United States). The lowest detection limit was 3.5 pg/mL with an intra-assay and inter-assay coefficient with less than 10% variation. Hs-CRP was measured by latex agglutination. ABI and CAVI were utilized to determine vascular complications using a portable ultrasonography-based machine (VaSera VS-200; Fukuda-Denshi Company, Tokyo, Japan). ABI was calculated by the highest systolic pressure of the foot of that side/average of the highest pressure from both arms, while the CAVI score was analyzed by the machine. ABI scores < 0.9 and 0.91–0.99 indicate peripheral arterial disease and borderline, respectively, while ABI at 1–1.4 and >1.4 are normal and non-compressible arteries, respectively [33]. CAVI scores < 8 and 8–9 are normal and are at risk for atherosclerosis, respectively, while CAVI > 9 indicates possible atherosclerosis [34]. Details regarding the data collection parameters are listed in Supplementary Table S1.

### 5.4. Statistical Analysis

We estimated the sample size based on the results of previous clinical trials. The sample size was calculated using total p-cresyl sulfate as the main variable and by assuming a power of 80% and a two-sided alpha of 5%. Allowing for a 10% dropout or withdrawal rate, we estimated that minimum of 20 patients per group was required to detect a statistically significant difference between the two arms.

All of the analyses adhered to the intention-to-treat principle. We described patient characteristics using mean ± (standard error; SE) for normally distributed or median (interquartile range; IQR) for non-normally distributed continuous variables. Categorical data were described as numbers and percentages. We compared patient characteristics using the chi-square, unpaired t tests, and Mann–Whitney U test as appropriate. The changes in the primary and secondary outcomes from baseline in each group were compared using a paired t test. Treatment effects on the variables among patients treated with sevelamer or calcium carbonate were examined using linear mixed-effects models for repeated measures over time, including data from the baseline, 12th week, and 24th week. A random intercept model was used with time coded as categorical factor. Differences between the groups were estimated by including in the interaction term between visit and treatment the model. Results were reported as estimated marginal mean with 95% confidence intervals (95% CI). A two-tailed *p*-value < 0.05 was considered significant. Data were analyzed using SPSS statistic version 22 and Stata version 15.

## Figures and Tables

**Table 1:** Baseline demographic, biochemical, and clinical data between sevelamer and calcium carbonate groups.

Parameters	Sevelamer (n = 20)	Calcium Carbonate (n = 20)	*p*-Value
Age (year)	59.8 ± 2.95	52.05 ± 3.49	0.07
BMI (kg/m^2^)	26.08 ± 1.17	26.94 ± 1.33	0.92
Cause of CKD (%)			0.40
Diabetic nephropathy Hypertensive nephropathy	12 (60%) 1 (5%)	8 (40%) 4 (20%)	
Chronic glomerulonephritis ADPKD	1 (5%) 1 (5%)	2 (10%) 1 (5%)	
Unknown	5 (25%)	5 (25%)	
BUN (8–20 mg/dL)	69.1 ± 3.66	68.43 ± 4.60	0.91
Creatinine (0.6–1.2 mg/dL)	6.51 ± 0.69	6.03 ± 0.62	0.68
eGFR (mL/min/1.73 m^2^)	9.79 ± 1.50	11.08 ± 2.09	0.61
CKD stage			0.34
4 (eGFR 15–30 mL/min/1.73 m^2^)	3 (15%)	1 (5%)	
5 (eGFR < 15 mL/min/1.73 m^2^)	17 (85%)	19 (95%)	
Proteinuria (0–0.15 g/day)	2.12 ± 0.06	2.45 ±0.05	0.58
Urine sodium (mEq/day)	136.13 ± 4.42	167.02 ± 8.01	0.48
Dietary protein intake (g/kg/day)	0.72 ± 0.06	0.70 ± 0.05	0.90
Calcium (8.6–10.2 mg/dL)	8.96 ± 0.18	8.88 ± 0.20	0.83
Phosphate (3–4.5 mg/dL)	5.50 ± 0.17	5.39 ± 0.10	0.62
iPTH (15–65 pg/mL)	336.5 ± 42.22	303.78 ± 58.27	0.64
FGF23 (pg/mL)	47.19 (91.08)	61.50 (83.73)	0.64
25 OH vitamin D (20–40 ng/mL)	25.85 ± 2.61	26.68 ± 4.49	0.87
LDL cholesterol (mg/dL)	112.89 ± 13.18	89 ± 7.95	0.15
HDL cholesterol (mg/dL)	40.97 ± 3.24	42.68 ± 3.39	0.72
Albumin (3.4–5.4 g/dL)	3.94 ± 0.09	3.87 ± 0.08	0.67
Hemoglobin (12–15.5 g/dL)	9.76 ± 0.24	9.44 ± 0.27	0.38
Hs-CRP (0–3 mg/L)	0.81 (0.96)	0.8 (2.75)	0.59
ABI (1–1.4)	1.1 ± 0.02	1.09 ± 0.03	0.52
CAVI (<8)	8.36 ± 0.27	7.47 ±0.37	0.08
LVEF (≥50%)	62.48 ± 3.84	65.52 ± 2.35	0.54
LVMI (≤115 g/m^2^ for male and ≤95 g/m^2^ for female)	123.19 ± 9.94	124.24 ± 10.18	0.94
LVH (%)	17 (85%)	16 (80%)	0.81
Serum p-cresyl sulfate (mg/L)	10.75 ± 2.20	8.75 ± 1.26	0.45
Serum indoxyl sulfate (mg/L)	17.97 ± 2.55	16.16 ± 2.49	0.62

Data are presented as mean ± standard error (SE) or median (interquartile range). Abbreviations: ABI—ankle–brachial index, ADPKD—autosomal dominant polycystic kidney disease; BMI—body mass index; BUN—blood urea nitrogen; CAVI—cardio–ankle vascular index; CKD—chronic kidney disease; eGFR—estimated glomerular filtration rate; FGF23—fibroblast growth factor 23; HDL—high density lipoprotein; hs-CRP—high sensitivity C reactive protein; iPTH—intact parathyroid hormone; LDL—low density lipoprotein; LVEF—left ventricular ejection fraction; LVH—left ventricular hypertrophy; LVMI—left ventricular mass index.

**Table 2:** Laboratory parameters during the follow-up period in two treatment groups.

	Sevelamer			Calcium Carbonate			*p*-Value (between Groups)
Parameters	Baseline	12 weeks	24 weeks	Baseline	12 weeks	24 weeks	
CKD-MBD parameters							
Calcium (mg/dL)	8.96 ± 0.18	8.89 ± 0.17	8.65 ± 0.28	8.88 ± 0.20	9 ± 0.27	8.85 ± 0.40	0.42
Phosphate (mg/dL)	5.50 ± 0.17	4.85 ± 0.22	5.4 ± 0.44	5.39 ± 0.10	5.14 ± 0.23	6.05 ± 0.70	0.36
iPTH (pg/mL)	336.5 ± 42.22	319.85 ± 45.83	357.3 ± 72.99	303.78 ± 58.27	317.72 ± 60.27	339.33 ± 79.02	0.75
FGF23 (pg/mL)	47.19 (91.08)	53.18 (230.28)	57.20 (122.27)	61.50 (83.73)	180.63 (212.11 *)	106.07 (208.4 *)	0.01
Lipid profiles							
LDL cholesterol (mg/dL)	112.89 ± 13.18	72.55 ± 7.28 *	72.37 ± 12.74 *	89 ± 7.95	83.44 ± 4.33	77.91 ± 7.81	0.04
HDL cholesterol (mg/dL)	40.97 ± 3.24	42.58 ± 3.70	45.21 ± 4.48	42.68 ± 3.39	44.67 ± 3.17	41.1 ± 4.05	0.58
Nutrition parameters							
Albumin (g/dL)	3.94 ± 0.44	4.68 ± 0.81	3.85 ± 0.15	3.87 ± 0.35	3.98 ± 0.09	3.89 ± 0.12	0.91
Dietary protein intake (g/kg/day)	0.72 ± 0.06	0.68 ± 0.07	0.71 ±0.04	0.70 ± 0.05	0.72 ±0.04	0.68±0.04	0.39
Renal parameters							
BUN (mg/dL)	69.1 ± 3.66	73 ± 5.02	75.93 ± 6.64	68.43 ± 4.60	82.55 ± 6.22	91.41 ± 6.84	0.21
Creatinine (mg/dL)	6.51 ± 0.69	6.95 ± 3.22	8.89 ± 1.65	6.03 ± 0.62	7.89 ± 4.13	10.52 ± 1.90	0.26
eGFR (mL/min/1.73 m^2^)	9.79 ± 1.50	8.55 ± 1.12 *	7.73 ± 1.04 *	11.08 ± 2.09	9.58 ± 2.42 *	6.25 ± 0.88 *	0.99
Proteinuria (g/day)	2.12 ± 0.06	2.34 ± 0.62	2.56 ± 1.39	2.45 ±0.05	2.61 ± 1.02	2.68 ± 1.63	0.78
Anemia and inflammatory markers							
Hemoglobin (g/dL)	9.76 ± 0.24	9.55 ± 0.27	9.28 ± 0.22	9.44 ± 0.27	9.34 ± 0.31	8.84 ± 0.52	0.56
Hs-CRP (mg/L)	0.81 (0.96)	0.98 (2.37)	1.03 (5.95)	0.8 (2.75)	0.96 (1.89)	2.23 (3.55)	0.64
Vascular stiffness parameters							
ABI (1–1.4)	1.1 ± 0.02	N/A	1.1 ± 0.02	1.09 ± 0.03	N/A	1.1 ± 0.03	0.57
CAVI (<8)	8.36 ± 0.27	N/A	8.25 ± 0.31	7.47 ± 0.37	N/A	7.54 ± 0.37	0.42

Data are presented as mean±SE, median (IQR). Abbreviations: ABI—ankle–brachial index; BUN—blood urea nitrogen; CAVI—cardio–ankle vascular index; eGFR—estimated glomerular filtration rate; FGF23—fibroblast growth factor 23; HDL—high density lipoprotein; hs—CRP-high sensitivity C reactive protein; iPTH—intact parathyroid hormone; LDL—low density lipoprotein.* *p* < 0.05 within a group versus baseline.

## Data Availability

The datasets generated and/or analyzed during the current study are not publicly available, but are available from the corresponding author on reasonable request.

## Supplementary Materials

The following are available online at https://www.mdpi.com/article/10.3390/toxins13100688/s1, Table S1: Data Collection.
