# Supplementary material for: Protein-Bound Uremic Toxins Lowering Effect of Sevelamer in Pre-Dialysis Chronic Kidney Disease Patients with Hyperphosphatemia: A Randomized Controlled Trial
Source: Toxins (Basel). 2021 Sep 27;13(10):688. doi: 10.3390/toxins13100688 (PMC8539528; doi:10.3390/toxins13100688)
Supplement: Supplementary file 1 [file toxins-13-00688-s001.zip › toxins-1374864-supplementary.pdf]

toxins

Supplementary Information

Protein-bound Uremic Toxins Lowering Effect of Sevelamer in Pre-dialysis Chronic Kidney Disease Patients with Hyperphosphatemia: A Randomized Controlled Trial

Table S1. Data collection.

	Screening	Baseline	6 weeks	12 weeks	24 weeks
Inclusion and Exclusion criteria	✓				
Demographic data	✓				
Cause of chronic kidney disease	✓				
Underlying disease	✓				
Laboratory data	✓	✓	✓	✓	✓
24-hour urine	✓	✓	✓	✓	✓
p-cresyl sulfate		✓		✓	✓
Indoxyl sulfate		✓		✓	✓
Fibroblast growth factor 23		✓		✓	✓
High sensitivity C reactive protein		✓		✓	✓
Lipid profiles		✓		✓	✓
Parathyroid hormone		✓		✓	✓
Cardio-ankle vascular index		✓			✓
Echocardiography		✓			
Renal replacement therapy status		✓	✓	✓	✓
Cardiovascular event		✓	✓	✓	✓
Dead or alive status		✓	✓	✓	✓
Adverse events		✓	✓	✓	✓
